# South African emerging adults’ capacity for resilience in the face of COVID-19 stressors

**DOI:** 10.1177/13591053231208620

**Published:** 2023-11-16

**Authors:** Kate Cockcroft, Mike Greyling, Ansie Fouché, Michael Ungar, Linda Theron

**Affiliations:** 1 School of Human and Community Development at the University of the Witwatersrand, South Africa; 2 Data Management and Statistical Analysis, South Africa; 3 United Arab Emirates University, UAE; 4 North-West University, South Africa; 5 Dalhousie University, Canada; 6 University of Pretoria, Lynnwood Avenue, Pretoria 0002, South Africa

**Keywords:** COVID-19, cross-sectional study, emerging adults, resilience, South Africa

## Abstract

Little is known about resilience responses to COVID-19 stressors from emerging adults in minority world contexts. In this cross-sectional study, we explored the association between self-reported COVID-19 stressors and capacity for resilience in 351 emerging adults (Mean_age_ = 24.45, SD = 2.57; 68% female) who self-identified as Black African. We were interested in whether age, gender and neighbourhood quality influenced this association. The main findings were that higher pandemic stress was associated with a greater capacity for resilience. Older participants showed higher levels of resilience, while there was no gender difference in this regard. Those who perceived their neighbourhoods as being of a good quality also showed greater capacity for resilience, despite all participants residing in disadvantaged communities. The theoretical and practical implications of these results are considered.

## Introduction

While emerging adults (aged 18–29 years; Arnett, 2000) are at lesser risk of COVID-19 severe illness and mortality, the negative impact of the pandemic on mental health and well-being is reportedly higher among this cohort than other age groups (Angela et al., 2022; Glowacz and Schmits, 2020; O’Connor et al., 2021). For example, meta-analyses show that youth risk for depression increased during the COVID-19 pandemic (Racine et al., 2021; Wang et al., 2021), a trend also evident in South Africa, where the current study is located (Haag et al., 2022; Mudiriza and De Lannoy, 2020).

The lingering aftereffects of the COVID-19 pandemic (e.g. unemployment, reliance on parents for financial and/or social support, postponement of significant life events) have impacted the transition to adulthood for emerging adults whose developmental challenges include navigating their independence from their families, completing higher education, seeking and securing work and/or forming meaningful relationships with long-term partners (Billari and Liefbroer, 2010). These uncertainties and challenges appear to have resulted in increased stress and mental health issues among this age group (Glowacz and Schmits, 2020; O’Connor et al., 2021). In particular, youth from poor socio-economic circumstances in low- and middle-income countries, like South Africa, are vulnerable to mental health challenges, yet have limited access to appropriate mental health services (Stelmach et al., 2022). Therefore, understanding those factors and processes that might enable effective coping and adaptation (or capacity for resilience) in youth is an essential contribution towards prevention/early intervention in the absence of mental health/psychological services (Luthar et al., 2000).

Resilience can buffer the adverse effects of stressors on mental health and is conceptualised as a capacity that enables quick recovery from stress, flexible adaptation to new situations, and learning positive lessons or ‘bouncing back’ from adversity (Yıldırım et al., 2022). Capacity for resilience in the face of the COVID-19 pandemic would therefore enable an easier and more positive reconciliation of the developmental challenges of emerging adulthood.

The multisystemic approach to resilience argues that factors at all systemic levels (individual, interpersonal, institutional and ecological) contribute to mental health outcomes (Masten et al., 2021; Ungar and Theron, 2020). Most is known about the individual factors (e.g. social skill, positive meaning making, agency) that contribute towards resilience (Luthar et al., 2000), or how these interact dynamically with interpersonal/social factors (e.g. caring family or supportive peers) to produce resilience (Masten et al., 2021), while least is known about institutional and ecological contributions (Ungar and Theron, 2020). Institutional resources include effective schools and religious institutions, mental health services and governmental facilities, while ecological resources comprise neighbourhood factors such as green spaces and safe recreational facilities (Theron et al., 2023). The multisystemic approach views individual factors as complemented by interpersonal, institutional and ecological ones to support positive outcomes under high stress exposure (Masten et al., 2021). These protective factors and processes (PFPs) can be mobilised in the event of adversity, stressors and challenges to ensure greater capacity for resilience and the maintenance of well-being (Liu et al., 2017). In this study, we were interested in those PFPs drawn on by emerging adults during the COVID-19 pandemic.

A few studies have explored PFPs that contributed to resilient coping during the pandemic in minority-world contexts (i.e. wealthier, developed nations, constituting a small percentage of the global population; Pence, 1998). These studies tend to focus on PFPs at an individual or interpersonal level, with few exploring resources at the institutional or ecological levels. For example, Kimhi et al. (2020) investigated the relationship between resilience, demographic variables (including age and gender), distress and perceived danger during the pandemic in 1346 Jewish Israelis (Mean_age_ = 42 years; SD = 16.35). Resilience was conceptualised as individual, interpersonal, community (cohesion and provision of support), institutional and national resources (e.g. confidence and trust in government institutions and systems), while ecological resources were not assessed. Community, institutional and national factors were not predictive of distress symptoms. This may be because COVID-19 poses primarily a personal health threat. Older and male participants from larger communities reported significantly lower distress and danger than did younger and female participants from smaller communities.

Similarly, Panzeri et al. (2021) investigated the individual and institutional (societal and community) factors related to resilience in 1034 Italian adults (Mean_age_ = 49.9 years; SD = 16.2) during the first pandemic wave. Community resources included participation, support and cohesion with one’s community (including religious institutions), while societal resources referred to social policies, access to and quality of social and health services. Factors at the individual (being older and male) and societal (but not the community) levels contributed to resilient outcomes in this sample. However, participants in both studies were considerably older than our sample, and age may mediate resilient functioning (Kimhi et al., 2020; Panzeri et al., 2021). With age and life experience, a repertoire of coping resources is developed that enable the navigation of challenges and stressors more easily than in younger years (Charles, 2010).

These studies suggest that, in majority-world contexts in addition to age, gender predicts coping with stress in the context of a pandemic, with better outcomes for males (Kimhi et al., 2020; Panzeri et al., 2021). Women, irrespective of their age, may have borne additional stress during the pandemic due to an increase in household and child-care responsibilities (Panzeri et al., 2021).

None of the above studies explored the potential contribution of resources at the level of physical ecology. Access to green spaces and positive perceptions of neighbourhood quality are associated with various mental health outcomes, but this has not been extensively researched in studies of capacity for resilience to COVID-19 stressors (Oswald et al., 2021). Neighbourhood quality includes feeling safe enough to engage in outdoor activities that may enhance mental health, social cohesion, extent of ecological dilapidation of the environment and signs of illegal activity (e.g. drinking in public, drug use/sales). During the COVID-19 pandemic, youth in the USA with access to safe outdoor spaces showed higher resilience during this time (Kilgore, 2020), while Australian youth associated unintentional or intentional contact with nature (e.g. outdoor gardens) with a capacity to flourish during the pandemic (Oswald et al., 2021). Two other studies implied a contribution by physical ecological resources when reporting on adaptive coping with COVID-related stressors. For example, Suhail et al. (2021) reported a young adult participant’s account of walking their dog and enjoying nature. Similarly, Golemis et al. (2022) suggested that access to outdoor spaces that facilitated sporting activity also enabled Greek youths’ capacity to deal positively with pandemic stressors. Consequently, quality of neighbourhood may be an important resource for youth facing COVID-19 stressors, although the role may be indirect by providing a safe and suitable environment for physical exercise.

The above studies detail the research into the PFPs that support the mental health resilience of emerging adults in minority-world contexts under the stressors of a pandemic, but this has not been extensively researched in majority-world contexts (continents that comprise most of the world’s population, e.g. Africa and South America; Pence, 1998). Haag et al. (2022) explored interpersonal PFPs drawn on by 233 South African youth (Mean_age_: 19.6 years) from resource-constrained communities. While COVID-related challenges predicted symptoms of anxiety and depression, positive experiences (e.g. time spent with family or recreational activities) ameliorated these symptoms. Another survey of 5693 South African youth (18–35 years) found that employment and/or caring for family corresponded with better mental health despite COVID-related challenges (Mudiriza and De Lannoy, 2020). These studies largely echo those from the minority-world in that personal and interpersonal PFPs were identified as key, but did not identify PFPs beyond these levels.

Given the limited attention to understanding the capacity for resilience in emerging adults from contexts such as Africa during the COVID pandemic (Rother et al., 2022; Theron et al., 2023), we were interested in whether the individual/personal qualities of age and gender played a similar role to that found in other studies. We also extended the focus to the physical ecology, an under-researched resilience resource. Following Ungar (2019), a focus was on which PFPs matter more at varying levels of pandemic stress exposure, and so we sought to understand how high, medium and low subjective experiences of pandemic stress are related to capacity for resilience in South African emerging adults from disadvantaged backgrounds. Attention to the PFPs that add differential value to the capacity for resilience of African youth is particularly valuable, given predictions that this will be the world’s largest population by 2050 and apprehension that this ‘youth dividend will not be realised unless this population’s resilience is nurtured’ (Theron, 2020: 1).

## Methods

### Participants and sampling

Participants were 351 emerging adults (Mean_age_ = 24.45, SD = 2.57; 68% female), who self-identified as Black African. Inclusion criteria were age between 18 and 29 years; English literate; resident in a disadvantaged (e.g. high density, poor infrastructure, low socioeconomic status) community in Gauteng, South Africa; personal experience of COVID-19-related stress (e.g. disrupted education/future plans; financial strain; separation from/loss of someone significant); identify as coping despite these stresses and disadvantaged community residence (e.g. engaged in further education/training; employed/actively seeking employment; contributing to household (e.g. child-minding)).

Recruitment occurred through social media, advertisements and staff at youth-focused non-government organisations. Prospective participants who met the inclusion criteria texted the study number and trained research assistants (RAs; psychology/social work graduates) responded telephonically. They explained the study’s purpose and checked that participants met the eligibility criteria. If participants were willing to participate, RAs emailed information and consent letters. Once these were returned, online links were emailed to participants.

### Measures

All measures were completed electronically (via a participant-specific link to Opinio). Sample items are available in the Supplemental Files.

#### Capacity for resilience

As in prior resilience studies (e.g. Bonanno, 2004, 2021; Theron et al., 2023), this was operationalised as the perception of resilience-supporting resources from the combined scores on two measures (CYRM-28 and RRM), and mental health, measured by absence of depressive symptoms (BDI-II).

##### The Rugged Resilience Measure (RRM; Jefferies et al., 2022)

Ten items tap individual resilience, focusing on internal, psychological protective factors. Participants rate their agreement with statements on a Likert scale ranging from 1 (not at all) to 5 (a lot). Scores (ranging between 10 and 50) are summed, with higher scores reflecting higher resilience. The RRM was highly reliable (Cronbach’s α = 0.931).

##### The Child and Youth Resilience Measure (CYRM-28)

This comprises 28 items, divided into three subscales, namely individual resilience resources (personal skills, e.g. problem solving, cooperation, social skills, peer support and awareness of strengths); relational resilience resources (bonds with parents/guardians/primary caregivers, including material and psychological caregiving); and contextual resources that facilitate connections to culture, religious and spiritual beliefs and practices, and engagement with, and relevance of, one’s educational institution. A few wording changes were made in keeping with the equivalent items in the adult version to make the CYRM-28 more suitable for emerging adults. Summed items yield a total resilience score. Scores were also summed per subscale with higher scores representing higher resilience. The CYRM-28 was very reliable (Cronbach’s α = 0.941) (Ungar and Liebenberg, 2011).

##### The Beck Depression Inventory-Second Edition (BDI-II)

Since depression has been negatively correlated with resilience (Bonanno, 2004, 2021), we included BDI-II (Beck et al., 1996) scores in the measurement of resilience. Participants self-report listed symptoms of depression in the preceding 2 weeks. There are 21 items, each comprising four statements related to increasing symptom severity, scored 0–3, for example, ‘0 = I do not feel sad; 1 = I feel sad much of the time, 2 = I am sad all the time; 3 = I am so sad or unhappy that I can’t stand it’. Scores are summed, with totals between 0 and 13 denoting no/minimal depression symptoms; 14–19 indicative of mild symptoms; 20–28 suggesting moderate symptoms; and 29–63 signalling severe symptoms. The BDI-II was extremely reliable (α = 0.935).

#### Independent variables

##### The Pandemic Stress Questionnaire

This taps subjective experiences of exposure to stressful events due to COVID-19. It comprises 25 items divided into six sub-scales measuring different aspects of pandemic stress, namely, general life disruption (six items), interpersonal stress (five items), financial stress (three items), educational/professional goals (three items), own health (five items) and health of others (three items). Participants are first asked whether they have had any of these experiences because of the COVID-19 pandemic. This is followed by a Likert-type style severity rating, ranging from 1 (Not at all) to 5 (Extremely bad), with higher scores indicating higher levels of pandemic-related stress. The questionnaire was very reliable (α = 0.908) (Kujawa et al., 2020).

##### Perception of Neighbourhood Scale

This 13-item scale assesses perception of the quality of one’s neighbourhood and communities through a set of statements on a Likert-type scale ranging from 1 (Always False) to 4 (Always True). Statements relate to social cohesion, sense of safety, ecological degradation and illegal activity (e.g. drug sales). Scores are summed and higher scores indicate more positive perceptions. The scale’s reliability was good (Cronbach’s α = 0.786) (Ruchkin et al., 2004).

### Data processing

A Bayesian four-way Analysis of Variance (ANOVA) explored the relationship between capacity for resilience and perceived pandemic stressors, with age, gender and neighbourhood quality as moderators. The posterior distributions generated by Bayesian analysis speak directly to the plausibility of the hypothesis under investigation, rather than the likelihood of the observed data (as in frequentist statistics). Bayesian approaches also allow for multiple models to be considered simultaneously and ranked according to the probability that they are correct. The analysis does not choose an outcome based on significance criteria and so is less prone to false positives due to the multiple comparison problem (Vuong et al., 2020). The dependent variable was a composite resilience score obtained from the factor analysis of the resilience measures. The independent variables were scores on the Pandemic Stress Questionnaire (PSQ), the Perception of Neighbourhood Scale (CRPN), age and gender. Since the former two variable distributions had some extreme values, it was difficult to justify a-priori that the results were linear across the range. Subsequently, they were grouped into three categories (low, medium and high) by splitting at the 33.3% and 66.6% percentiles. This approach loses some information, but strengthens the conclusions by reducing the risk of outliers and non-linear relationships. Age was split into two categories (younger: 18–24 and older: 25–29), while gender responses yielded two categories (male/female). The Bayesian approach considers all possible combinations of these variables (subject to the constraint that interaction terms must have the precursor terms). The selected prior distribution made all models equally likely, allowing the data to direct which model was the most probable.

### Ethics

Data collection occurred between June and December 2021, when the South African government implemented strict lockdown restrictions and public health measures to restrict the spread of COVID-19). During this time, in-person research was prohibited so data collection was virtual/telephonic.

Ethical clearance was granted by the Faculty of Health Sciences Research Ethics Committee and Faculty of Education Ethics Committee, University of Pretoria (clearance number: UP17/05/01). Participants provided written and verbal consent prior to participation. They received 50 MB of data to complete the measures and compensation for their participation (approximately $10 supermarket voucher).

## Results

Data was analysed with R (R Core Team, 2021) and JASP (JASP Team, 2023). Table 1 shows the means, standard deviations and score ranges for the measures in this study.

**Table 1.** Means, standard deviations (SD) and ranges of study variables.

	Mean (SD)	Range
Beck Depression Inventory-II	7.82 (9.47)	0–63
Rugged Resilience Measure Total	44.36 (6.36)	10–50
Child and Youth Resilience Measure-28 (CYRM Total)	120.39 (19.68)	28–140
CYRM-28 Individual	40.43 (7.73)	11–55
CYRM-28 Relational	30.00 (5.91)	7–35
CYRM-28 Contextual	42.93 (7.38)	10–50
Perception of Neighbourhood Scale	32.84 (5.44)	13–52
Pandemic Stress Questionnaire	56.22 (29.47)	25–125

Depressive symptoms tended to be low (minimal to mild) in the sample, with a range of levels of resilience reported. There was considerable variation in the sample’s perceived stress associated with the pandemic and their perceptions of their neighbourhoods, despite all residing in disadvantaged communities.

### Correlations

As expected, greater capacity for resilience (CYRM-28 and RRM scores) were significantly, negatively correlated with depression symptoms (BDI-II scores). The CYRM-28 subscales (tapping individual, relational and contextual resilience resources) were all strongly and positively inter-correlated (Table 2).

**Table 2.** Correlations between study variables.

Variable	1	2	3	4	5	6	7	8	9
1. Depression (BDI-II)									
2. Child and Youth Resilience Measure (CYRM-28)	−0.737**								
3. CYRM-28 Individual	−0.710**	0.956**							
4. CYRM-28 Relational	−0.688**	0.941**	0.849**						
5. CYRM-28 Contextual	−0.712**	0.963**	0.899**	0.880**					
6. Gender	−0.141**	0.030	0.030	0.019	0.048				
7. Age	−0.190**	0.242**	0.284**	0.199**	0.219**	0.080			
8. Pandemic Stress Questionnaire	−0.489**	0.580**	0.556**	0.528**	0.584**	−0.024	0.209**		
9. Perception of Neighbourhood Scale	−0.409**	0.447**	0.392**	0.427**	0.481**	0.080	0.211**	0.267**	0.324**

* *p* < 0.05. **p < 0.01.

A factor analysis indicated that a single factor explained 74% of the variance between the variables operationalising capacity for resilience (CYRM-28, RRM and BDI-II), with the lowest absolute correlation at 0.76 (Table 3). Thus, these variables formed a single coherent structure representing capacity for resilience and this factor was used in subsequent analyses.

**Table 3.** Factor loadings of the capacity for resilience measures.

Variable	Factor 1
Depression (BDI II)	−0.76
Rugged Resilience Measure	0.83
Contextual resilience resources (CYRM-28)	0.87
Relational resilience resources (CYRM-28)	0.90
Personal resilience resources (CYRM-28)	0.92

A Bayesian four-way Analysis of Variance (ANOVA) using JASP (JASP Team, 2023) examined the relationship between capacity for resilience and perceived pandemic stressors. The potential moderating effects of age, gender and neighbourhood quality were also considered. The 10 models with the highest posterior probabilities are in Table 4. The most probable model has a posterior probability of 0.47. All of the most probable models include the main effects of perceived pandemic stress and neighbourhood quality. The main effects of age and gender are also frequent, although gender does not appear in the two most probable models. The Bayes factor comparisons indicate that there is moderate evidence that the best model is better than the second- and third-best models and strong evidence that it is better than all the others. Based on these results, the estimates for the main effect parameters were examined with particular focus on the variables of pandemic stress and perception of neighbourhood quality.

**Table 4.** Bayesian ANOVAs comparing the models.

Models	P(M)	P(M|data)	BF_M_	1/BF_10_	Error %
1. Neighbourhood + Pandemic Stress + Age					
2. Neighbourhood + Pandemic Stress	0.006	0.16	31.89	0.344	2.97
3. Neighbourhood + Pandemic Stress + Age + Gender	0.006	0.06	10.49	0.127	3.47
4. Neighbourhood + Pandemic Stress + Age + Gender + Neighbourhood × Gender	0.006	0.05	8.04	0.099	11.42
5. Neighbourhood + Pandemic Stress + Age + Neighbourhood × Age	0.006	0.04	7.40	0.091	3.42
6. Neighbourhood + Pandemic Stress + Age + Pandemic Stress × Age	0.006	0.03	4.66	0.058	3.49
7. Neighbourhood + Pandemic Stress + Age + Neighbourhood × Pandemic Stress	0.006	0.03	4.57	0.057	4.27
8. Neighbourhood + Pandemic Stress + Gender	0.006	0.03	4.36	0.055	3.13
9. Neighbourhood + Pandemic Stress + Age + Gender + Neighbourhood × Gender + Age × Gender	0.006	0.02	2.97	0.037	4.11
10. Neighbourhood + Pandemic Stress + Gender + Neighbourhood × Gender	0.006	0.02	2.82	0.036	19.82

**Figure 1.**
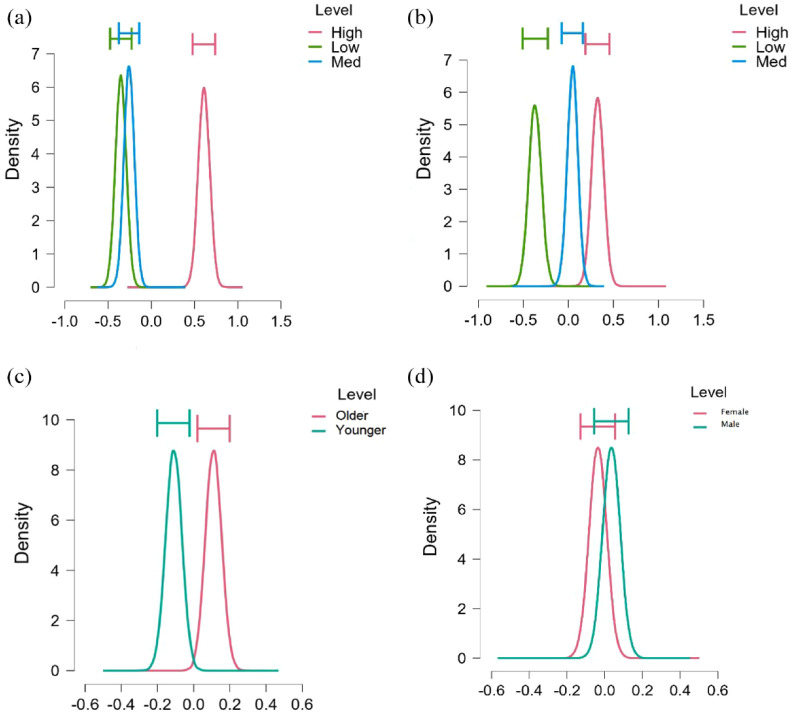
Credible intervals for parameter estimates.

The credible intervals for the parameter estimates are based on all the models weighted at their posterior probability (Figure 1(a)–(d)). The posterior distribution represents the updated probability distribution of the parameters of interest after incorporating both prior information and observed data. The parameters of interest in an ANOVA are the mean differences between groups. Each parameter reflects the difference between the overall mean and the mean for that group. A parameter estimate (*x* axis) greater than 0 indicates that group has a higher than average mean. Each graph shows the posterior distribution (probability density function, *y* axis) of the estimated parameter estimates, describing which values for a parameter have high probability (high values) and which have low probability. The area under any section of the curve is equal to the probability that the parameter lies in that part of the range. The section of the range where most of the area is contained allows the construction of a credible interval in which we have a high degree of belief that the parameter is contained within that interval.

In Figure 1(a), the three graphs represent the mean resilience for high, medium and low pandemic stress groupings (depicted in the legend). They indicate that greater capacity for resilience is associated with high levels of pandemic stress, but not with moderate or low levels. Most of the area for high pandemic stress is contained in the range (0.4; 0.8) on the *x* axis, while the credible ranges for low and medium pandemic stress are (−0.6; −0.2) and (−0.4; 0) respectively. There is a substantial difference in the position of the credible interval for high levels of pandemic stress as opposed to medium and low levels, while there is substantial overlap for the latter two levels of pandemic stress.

Figure 1(b) shows that higher levels of resilience are associated with a better neighbourhood perception (grouped into high, medium and low shown in the legend) and the credible intervals show minimal overlap. There is strong support for this pattern in the data. There is little overlap in the credible intervals shown in Figure 1(c), indicating strong evidence that mean resilience is higher in the older age group (25–29 years). The credible intervals in Figure 1(d) show significant overlap indicating that there is little support in the data for a gender difference. On average, men scored slightly higher on the resilience measures than women. This is consistent with the most probable model outcome which did not include gender.

## Discussion

This study investigated the associations between capacity for resilience and perceived pandemic stress during the COVID-19 pandemic in sample of South African emerging adults. We also considered the likelihood that neighbourhood quality, gender and/or age may influence these relationships.

There was strong evidence that participants who experienced the highest levels of pandemic stress also show the greatest capacity for resilience, while those experiencing moderate and low levels of pandemic stress demonstrated small differences in their levels of resilience. This extends findings with minority-world participants that adaptive functioning is a common response when exposed to significant risk, to both situations of pandemic stress and to majority-world participants (Bonanno, 2021). A review showed that 35%–65% of participants demonstrate resilience despite potentially traumatic events. In the review, resilience was characterised by minimal impairment, fleeting symptoms and a return to a stable trajectory of healthy functioning soon after the event (Bonanno, 2004). Our findings add support to the view that capacity for resilience in the face of stressors is one of our human capabilities, and that such resilience is also evident in emerging African adults exposed to substantial stress caused by a global pandemic (Bonanno, 2004, 2021; Masten et al., 2021). In contrast, those who experienced medium and low levels of pandemic stress have lower capacity for resilience, suggesting that stressors do not necessarily interfere with adaptive responding.

In contrast to studies that located PFPs contributing to resilience in youth during the pandemic at the individual and interpersonal levels (Kimhi et al., 2020; Panzeri et al., 2021), we found that the physical ecology also plays a positive role. Greater capacity for resilience was associated with a positively perceived neighbourhood, including a sense of social cohesion among neighbours, minimal perceived crime and a safe and physically attractive environment in which to engage in recreational activities and meet with friends. This corroborates similar associations with younger participants (Cameranesi et al., 2022; Refaeli and Achdut, 2022). This association was despite the fact that our participants resided in disadvantaged communities, which are characterised by many structural drawbacks (e.g. low quality housing, overcrowding, poor infrastructure, widespread poverty), inadequate service delivery and ongoing marginalisation (Haffejee and Levine, 2020). The differences between participants appeared to be at the perceptual level, with those who perceived their neighbourhoods more positively also showing greater capacity for resilience.

On an individual level, research from minority-world contexts suggests that the capacity for resilience in the face of the pandemic is associated with male gender and older age (Kimhi et al., 2020; Panzeri et al., 2021). Similarly, we found that, although our sample comprised emerging adults, the older group (25–29 years) showed higher capacity for resilience than the younger group (18–24 years). The older participants were more established in terms of their education and/or employment and so may have had more resources to draw on than their younger counterparts. In terms of gender, our results show a high probability of no gender differences in the association between COVID-related stress and capacity for resilience. The resilience literature is equivocal concerning gender differences in general, while research specific to COVID-19 stressors suggests that males fared better (Kimhi et al., 2020; Panzeri et al., 2021).

Our findings suggest that the greatest capacity for resilience is demonstrated by emerging adults who experienced the most pandemic-related stress, were older and resided in what they perceived as good quality neighbourhoods. This confirms research that adaptive responding to stressors is typical and that experience (linked to age) builds resilience (Bonanno, 2004, 2021). Our study also adds a new dimension to what is known about young adults’ responses to the pandemic, namely that responding to COVID-19 stressors is not purely an internal, personal process, but that the external environment can play a positive role.

Key implications of the study are that policies regarding urban planning and development should incorporate tangible ways in which the quality of a neighbourhood can be developed and improved, which may contribute towards adaptive coping. On a clinical level, interventions to build resilience may be most beneficial if they enable access to a repertoire of personal and contextual resources that could be drawn on in future difficult life circumstances. Finally, future follow-up studies should include a qualitative exploration to understand why the neighbourhood perceptions of those participants with greater capacity for resilience were positive, and how these differed from those with less capacity for resilience.

A few study limitations deserve mention. First, the cross-sectional nature of the data allowed observations of associations among variables but not inferences about potential causal relationships. Secondly, this study reports on early adaptive responses and these might differ from those in later stages of the pandemic.

## Conclusion

Most studies on resilience during COVID-19 explored risk for poor outcomes rather than capacity for resilience (e.g. Buizza et al., 2022; Elharake et al., 2023; Lee et al., 2022; Wang et al., 2021). Although the COVID-19 pandemic appears to be over, we may face other such events (Michie and West, 2021). Understanding what enabled young people’s resilience to COVID-19 stressors will prepare us for future pandemics, and pandemic-like situations. This is important since emerging adults were at greatest risk for impaired mental health during the pandemic compared to other age groups (Racine et al., 2021). Supporting younger people and ensuring safe and attractive physical environments may help to ease the effects of future world crises.

## Supplemental Material

sj-docx-1-hpq-10.1177_13591053231208620 – Supplemental material for South African emerging adults’ capacity for resilience in the face of COVID-19 stressors Supplemental material, sj-docx-1-hpq-10.1177_13591053231208620 for South African emerging adults’ capacity for resilience in the face of COVID-19 stressors by Kate Cockcroft, Mike Greyling, Ansie Fouché, Michael Ungar and Linda Theron in Journal of Health Psychology
