# Supplementary material for: South African emerging adults’ capacity for resilience in the face of COVID-19 stressors
Source: J Health Psychol. 2023 Nov 16;29(6):522–33. doi: 10.1177/13591053231208620 (PMC11075409; doi:10.1177/13591053231208620)
Supplement: sj-docx-1-hpq-10.1177_13591053231208620 – Supplemental material for South African emerging adults’ capacity for resilience in the face of COVID-19 stressors [file sj-docx-1-hpq-10.1177_13591053231208620.docx]

**Supplemental Files**

Example items for measures used in the study (excluding the Beck Depression Inventory-II which is copyright protected):

***The Rugged Resilience Measure*** (Jefferies et al., 2021).

| When faced with difficulties, I rise to the challenge | Not at all | A little | In-between a little and quite a lot | Quite a lot | A lot |
| --- | --- | --- | --- | --- | --- |
| I find solutions to problems I encounter | Not at all | A little | In-between a little and quite a lot | Quite a lot | A lot |

***The Child and Youth Resilience Measure*** (Ungar & Liebenberg, 2011).

| I have people I can respect in my life. | Not at all | A little | In-between a little and quite a lot | Quite a lot | A lot |
| --- | --- | --- | --- | --- | --- |
| I feel I belong in my community. | Not at all | A little | In-between a little and quite a lot | Quite a lot | A lot |
| I know my own strengths/what I am good at | Not at all | A little | In-between a little and quite a lot | Quite a lot | A lot |

***The Pandemic Stress Questionnaire*** (Kujawa et al., 2020).

| I was unexpectedly separated from family, friends, or others close to me because of the coronavirus pandemic (e.g., due to moves or travel restrictions). | Yes  No  *If yes: How bad was this event?*  *1=not at all bad*  *2 =slightly bad*  *3 =somewhat bad*  *4 = very bad*  *5 = extremely bad* |
| --- | --- |
| I had to take on additional responsibilities caring for others (e.g., children, other family members) due to the coronavirus pandemic. | Yes  No  *If yes: How bad was this event?*  *1=not at all bad*  *2 =slightly bad*  *3 =somewhat bad*  *4 = very bad*  *5 = extremely bad* |

***Perception of Neighbourhood Scale* (**Ruchkin et al., 2004).

| It is safe to walk alone in my neighbourhood/community after dark. | 1 = Always False  2 = Mostly False  3 = Mostly True  4 = Always True |
| --- | --- |
| Some people sell or use drugs in my neighbourhood/community. | 1 = Always False  2 = Mostly False  3 = Mostly True  4 = Always True |
